# US Cancer Screening Recommendations: Developments and the Impact of COVID-19

**DOI:** 10.3390/medsci10010016

**Published:** 2022-03-01

**Authors:** Adam Barsouk, Kalyan Saginala, John Sukumar Aluru, Prashanth Rawla, Alexander Barsouk

**Affiliations:** 1 Hillman Cancer Center, University of Pittsburgh, Pittsburgh, PA 15232, USA; adambarsouk@comcast.net; 2 Plains Regional Medical Group Internal Medicine, Clovis, NM 88101, USA; drsaginala@gmail.com; 3 Senior Research Associate, Beth Israel Deaconess Medical Center, Harvard Medical School, Boston, MA 02212, USA; jaluru@bidmc.harvard.edu; 4 Hospitalist, Parrish Medical Center, Titusville, FL 32796, USA; 5 Hematologist-Oncologist, Allegheny Health Network, Pittsburgh, PA 15212, USA; alexbarsouk@comcast.net

**Keywords:** COVID-19, cancer screening, USPSTF, pandemic, prevention

## Abstract

The USPSTF and ACS recommend screening for breast, cervical, colorectal, and lung cancers. Rates of cancer screening, diagnosis, and treatment decreased significantly in the US and other developed nations during the height of the COVID-19 pandemic and lockdown (April 2020) and have since recovered, although not to baseline levels in many cases. For breast cancer, the USPSTF recommends biennial screening with mammography for women aged 50–74, while the ACS recommends annual screening for women aged 45–54, who may transition to biennial after 55. Minority and rural populations have lower rates of screening and lower utilization of DBT, which offers superior sensitivity and specificity. Among 20 US health networks in April 2020, mammography rates were down 89.2% and new breast cancer diagnoses down by 50.5%. For cervical cancer, the USPSTF recommends cervical cytology every three years for women 21–65, or cytology+hrHPV co-testing every five years for women aged 30–65. Cervical cancer screening rates declined by 87% in April 2020 and recovered to a 40% decline by June 2020, with American Indians and Asians most severely affected. For colorectal cancer (CRC), the USPSTF and ACS recommend screening for ages 45–75, recently lowered from a starting age of 50. Most commonly-used modalities include annual FIT testing, FIT+DNA testing every three years, and colonoscopy every ten years, with shorter repeat if polyps are found. In the US, CRC screenings were down by 79–84.5% in April 2020 across several retrospective studies. Patient encounters for CRC were down by 39.9%, and a UK-based model predicted that 5-year-survival would decrease by 6.4%. The USPSTF recommends screening low dose CT scans (LDCTs) for ages 50–80 with a >20 pack-year smoking history who have smoked within the past 15 years. In April 2020, screening LDCTs fell by 72–78% at one US institution and lung cancer diagnoses were down 39.1%.

## 1. Introduction

**Figure 1:**
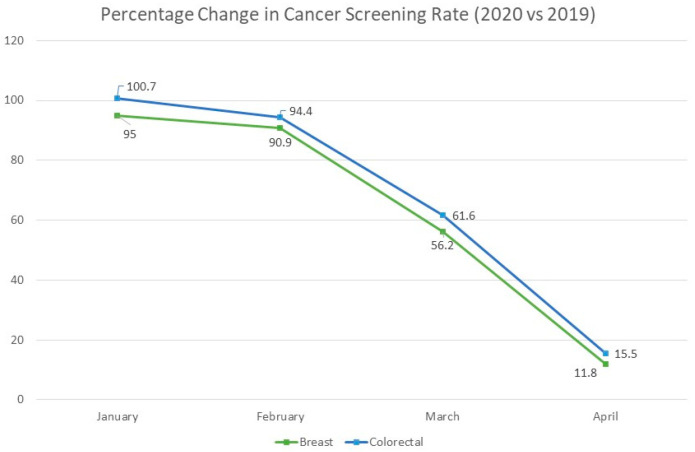
Decline in cancer screening rates during the peak of the COVID-19 pandemic. Data from “Effects of the COVID-19 Pandemic on Cancer-Related Patient Encounters.” *JCO Clin. Cancer Informatics* (2020).

Cancer screening in the US is an integral part of primary care and secondary prevention estimated to save hundreds of thousands of lives. Breast [1], cervical, colorectal [2], and lung cancer [3] each have evidence-based screening modalities that are recommended by the US Preventive Services Task Force (USPSTF) and American Cancer Society (ACS) for patients of certain ages and risks, while prostate cancer screening currently holds an equivocal recommendation [4]. The COVID-19 pandemic and ensuing lockdown, which reached its height in the US in April 2020, significantly decreased rates of screening, diagnosis, and treatment for these cancer types [5]. While screening and diagnosis rates are recovering, these missed opportunities for secondary prevention are projected to depress survival statistics for certain tumors for years to come [6], especially among vulnerable populations like Black, Hispanic, and rural Americans who have lower historic screening rates [7]. The purpose of this article is to examine the latest recommendations and technologies for cancer screening, demographic disparities in screening rates, and the estimated impact of COVID-19 on screening, diagnosis, and mortality (Figure 1).

## 2. Breast Cancer

Breast cancer (BC) is the most common neoplasm (besides skin cancer) and the second leading cause of cancer death among women in the US [8].

### 2.1. Current Breast Cancer Screening Recommendations

In 2016, the USPSTF issued a “B” grade recommendation that all women aged 50–74 years receive biennial screening mammography. Women aged 40–49 were issued a “C” grade recommendation, which advises them to come to an individualized decision with their provider on whether to pursue mammography. No grade “A” recommendations were issued [1]. In contrast, the ACS recommended in 2015 that women aged 45–54 receive screening mammography annually, while women 55 and older choose whether to receive annual or biennial mammography until life expectancy is below 10 years. Women aged 40–44 were advised to consider annual mammography with their providers. Women at high risk (*BRCA1* or *2* gene mutations, personal history or strong family history of breast cancer or tumor syndromes like Li–Fraumeni and Cowden, or chest radiation therapy at 10–30 years of age) are recommended to receive breast MRI and mammogram screening every year starting at age 30 [9].

Digital breast tomosynthesis (DBT), also known as 3D mammography, is considered the preferred imaging modality due to improved sensitivity, specificity, positive predictive value (PPV), negative predictive value (NPV) [10,11], and decreased compression of the breasts [12]. Breast MRI is utilized in adjunct in those with higher risk or denser breast tissue, which decreases the sensitivity of mammography [13]. Abbreviated breast MRI has been studied due to its reduced cost and comparable sensitivity, paving the way for broader utilization as a screening tool [14]. The Breast Imaging-Reporting and Data System (BI-RADS) developed by the American College of Radiology classifies findings on mammography, MRI, or ultrasound as BI-RADS 0: incomplete, 1: negative, 2: benign, 3: probably benign (<2% risk of malignancy), 4A: low suspicion of malignancy (2–9%), 4B: moderate suspicion for malignancy (10–49%), 4C: high suspicion for malignancy (50–94%), 5: highly suggestive of malignancy (>95%) and 6: biopsy-proven malignancy [11].

In the US, patients living in rural and low socioeconomic status zip-codes have lower rates of breast cancer screening [7]. African American and Hispanic women are significantly less likely to receive DBT, which is associated with superior breast cancer detection [15]. African American and non-English-speaking patients were also less likely to receive timely notification and follow-up of abnormal mammography results, which poses a higher risk for morbidity and mortality [16,17].

### 2.2. Impact of COVID-19 on Breast Cancer Screening

Lockdowns and public fear in the midst of the COVID-19 pandemic significantly decreased rates of breast cancer screening and diagnosis in the US. The rate of screening across 20 healthcare institutions dropped by 43.8% in March 2020 (as compared to March 2019) and by 89.2% in April 2020, constituting the largest decrease among cancer screening modalities, as published in JCO Clinical Cancer Informatics [5]. Several retrospective studies of US insurance databases and providers corroborated the decrease in screening and diagnosis. Data from the Clearinghouse database representing 5–7% of Medicare patients found that biopsy rates for breast cancer were reduced by 71% in April 2020 among US seniors (age > 65) [18]. Another retrospective study of 55 breast imaging centers across 27 US states found a 61.7% decline in breast imaging, a 20.5% decline in breast surgery, and a 39.9% decline in cancer genetics consultation from Feb 2020 to April 2020 [19].

Around the developed world, decreases in screening rates translated to decreased rates of diagnosis. A retrospective study based on the English national database found a 28% reduction in breast cancer diagnoses from January to July 2020 as compared to the same period in 2019 [20]. A retrospective study across 18 cancer centers in Austria found a 24% reduction in breast and gynecological cancer diagnoses in March 2020 and a 49% reduction in April 2020 [21]. Finally, a retrospective study of a hospital network in Macerata, Italy, found breast cancer diagnoses decreased by 26% in 2020 as compared to 2019 [22]. In the US, patients in the CCRN saw a 56.9% decrease in cancer-related appointments in April 2020, with a particular decline of 74% in new-incident cancers. Breast, melanoma, and prostate cancers were among the most affected, with 50.5% fewer new BC diagnoses [5].

**Figure 2:**
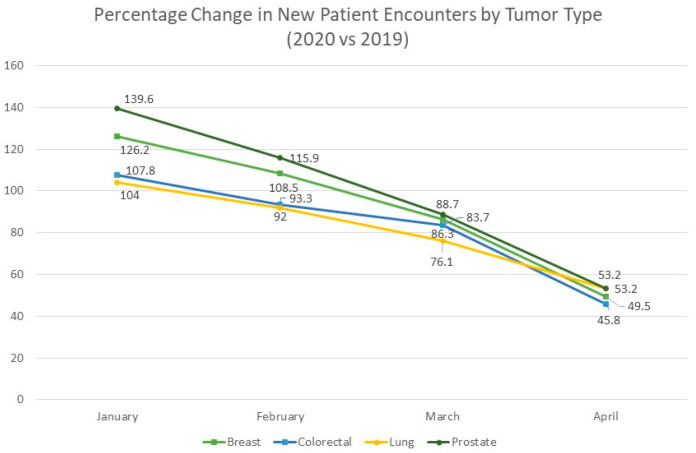
Decline in patient encounters during COVID-19 pandemic by cancer type. Data from “Effects of the COVID-19 Pandemic on Cancer-Related Patient Encounters.” *JCO Clin. Cancer Informatics* (2020).

Screening rates in the US have recovered since the initial days of the pandemic. Among select Medicare patients, while mammography rates were down 71% in April 2020, they were only down 31% in July [18]. Based on the Breast Cancer Surveillance Consortium data, screening and diagnostic mammography rates in April 2020 were 1.1% and 21.4% of their 2019 levels, respectively, but improved to 89.7 and 101.6% by July. Rates of screening in July of 2020 were 96.7% for Black women, 92.9% for White women, 72.7% for Hispanic women, and 51.3% for Asian women as compared to July 2019 [23]. While Asian and Hispanic women have historically reported lower rates of breast cancer screening, due to a multitude of structural and cultural factors, it seems the COVID-19 pandemic has presented new challenges to these populations that have prevented a return to baseline [24]. As the pandemic enters its second year, patients who remain hesitant to return for primary care will inevitably elapse the USPSTF’s biennial screening recommendations. The continued decline in new-incident cancer diagnoses suggests thousands of cases that are going undetected, which is likely to depress breast cancer prognosis in the coming decade (Figure 2).

## 3. Cervical Cancer

### 3.1. Current Cervical Cancer Screening Recommendations

As of 2018, the USPSTF has issued an “A” grade recommendation for cervical cytology (pap smear) screening every three years for women aged 21–29, with the choice to continue till 65 or begin combination high-risk human papilloma virus (hrHPV) testing plus cervical cytology every five years from ages 30–65. The USPSTF recommends against screening in women under 21, women older than 65 who have had three consecutive negative cytology results or two consecutive negative HPV tests, and women who have had a total hysterectomy with no history of high-grade precancerous lesions (CIN2–3) or cervical cancer. No grade “B” or “C” recommendations were issued [25]. As of 2021, the American College of Obstetrics and Gynecology (ACOG) and the Society of Gynecological Oncology (SGO) have both endorsed the USPSTF recommendations. As of 2020, the ACS recommends primary HPV testing as screening every 5 years from age 25–65, with co-testing or cytology (as recommended by the USPSTF) only offered when primary HPV testing is not available [26].

A study of the Swedish health registry found that from 2006–2017, the incidence of invasive cervical cancer was reduced by 88% for women receiving the quadrivalent HPV vaccine before the age of 17 and reduced by 53% in those vaccinated above 17 [27]. A prospective model in Switzerland found that the transition to the nine-valent HPV vaccine for 11–26 year old men and women would result in a further 24% reduction in cervical cancer incidence over the quadrivalent vaccine [28]. About 75.1% of US teens had received at least one dose of the HPV vaccine in 2020, which is projected to contribute to the continued decrease in cervical and head and neck cancer incidence [29].

### 3.2. Impact of COVID-19 on Cervical Cancer Screening

According to the National Breast and Cervical Cancer Early Detection Program (NBCCEDP), cervical cancer screening declined by 87% across the US in April 2020, with the most severe decline in the New York region (95%). The populations with the greatest declines were American Indian (98%) and Asian/Pacific Islander (92%). By June 2020, the screening rate recovered to 40% below the 5-year average [30]. A retrospective study of the Kaiser Permanente Southern California health system found that cervical cancer screening rates among women aged 21–29 in 2020 were decreased by 8% before the lockdown, decreased by 78% during the lockdown, and decreased by 29% after the lockdown was lifted (until 30 September 2020), as compared to the same time periods in 2019. For women aged 30–65, the reductions in screening were 3%, 82%, and 24%, respectively [31]. Another retrospective study out of Belgium found that the number of cervical cytology samples was reduced by roughly 59% in March 2020 and roughly 76% in April 2020 as compared to 2019 [32].

Unlike with CRC screening, self-sample cervical cancer screening is not yet approved in the US, although prior data have been compelling and the pandemic has prompted the NIH to spearhead an initiative. A *BMJ* meta-analysis found that while self-sample hrHPV assays were around 15% less sensitive and 2–4% less specific, they generated a 2.33× greater response rate on intention-to-treat analysis [33].

## 4. Colorectal Cancer

Colorectal cancer (CRC) is the fourth most common malignancy and the second leading cause of cancer mortality in the US [8].

### 4.1. Current Colorectal Cancer Screening Recommendations

As of 2021, the USPSTF has issued an “A” grade recommendation for colorectal cancer screening for adults aged 50–75 and a “B” grade recommendation for adults aged 45–49. Previous to 2021, patients aged 45–49 were not recommended regular screening. Adults 76–85 years of age have a “C” level recommendation, meaning that clinicians should selectively offer screening based on individual life expectancy and patient risk. Per the USPSTF, screening modalities include colonoscopy every 10 years, high-sensitivity guaiac fecal occult blood test (HSgFOBT) or fecal immunochemical test (FIT) every year, stool DNA-FIT every 1–3 years (tradename Cologuard), flexible sigmoidoscopy every 5 years, or flexible sigmoidoscopy every 10 years plus FIT every year [2]. The ACS likewise recommends regular screening for adults with average risk age 45–75, selective screening for those 76–85, and no screening for adults over 85. Certain high-risk factors may necessitate earlier screening than 45 per the ACS. Those with a family history of colorectal cancer should initiate screening 10 years prior to the age of diagnosis of their relative. Patients with a history of radiation to the abdomen or pelvis should begin screening 5 years after radiation or age 30, whichever comes first. Patients with inflammatory bowel disease require colonoscopies at least 8 years after diagnosis, repeated every 1–3 years. Likewise, patients with polyps removed during colonoscopy should repeat the test after at most 3 years, and possibly earlier if malignant or high-risk features are found on pathology [34].

Based on SEER 2014–2019 data, Black Americans are about 15% more likely to be diagnosed with CRC and 35% more likely to die of it. Black patients have lower rates of screening and consequently a higher risk of being diagnosed at a later stage [8]. A retrospective review of 11 studies of outreach strategies found that education on and follow-up of stool-based CRC screening modalities could increase screening rates among Black Americans by 1–13 times [35].

### 4.2. Impact of COVID-19 on Colorectal Cancer Screening

In a study of 20 US institutions published in *JCO Clinical Cancer Informatics*, CRC screening rates decreased by roughly 38.4% in March 2020 and 84.5% in April 2020 as compared to the same intervals in 2019. The study of Medicare patients in the Clearinghouse database found a near 79% decrease in CRC screenings in April 2020, which improved to a 33% decrease in July 2020 [5]. Data from the National Cancer Institute’s PROSPR Consortium predicted CRC screening rates dropped by 82% in 2020 [36].

In the *JCO Clinical Cancer Informatics* study, the number of patient encounters for CRC decreased by 39.9% in April 2020 as compared to 2019, a smaller decrease than that of breast (47.7%) or prostate cancer (49.1%) [5]. In the study of hospitals in Macerata, Italy, diagnosis of CRC was reduced by 62% in 2020 as compared to 2018–2019 [22]. A simulation based on the Canadian Cancer Registry predicted a 7% and 14% drop in CRC diagnoses with a 3- or 6-month halt in screenings, translating to a 10% or 19% increase in later-stage, symptomatic CRC cases, respectively [37].

Delays in screening likely translated to later diagnosis and poorer outcomes. A retrospective study of one hospital network’s data in Italy found that the rate of “high-risk adenomas” was significantly higher during the 2-month lockdown period as compared to 2019 (47% vs. 25%), as was the rate of colorectal cancers (8% vs. 1%), although selection bias for colonoscopies of more symptomatic patients likely occurred. Multiple regression analysis found that the lockdown period was an independent predictor of high-risk adenomas and CRC (HR = 2.2) [38]. A modeling study from the National Cancer Registry in England predicted a 6.4% decline in 5-year survival of CRC due to the COVID-19 lockdown and up to 16.6% more CRC deaths, as compared to only a 1% decline in survival for breast cancer [6].

Several studies of the NHS in the UK found that prioritization of HSgFOBT testing for 2-week-wait CRC patients improved the total number of patients screened [39]. and helped triage symptomatic patients [40]. Similarly, an initiative by the University of California Los Angeles (UCLA) health system to continue stool-based testing saw a decline in FIT testing of only 39.4%, as compared to a decline in colonoscopies of 88.6% from March to May 2020 [41]. These efforts mirror the success of pre-COVID initiatives to improve CRC screening via stool-based testing among the Black American community. Many US health plans such as OptumCare and Humana, as well as Federally Qualified Health Centers (FQHCs), have launched or expanded programs to send FIT kits to patients due for screening annually. Cologuard, the combined DNA/FIT test which is recommended every 3 years, has also increased market share, although the cost to payers remains a barrier (in 2020, Medicare paid $16 for a FIT test and $509 for the Cologuard test) [42].

## 5. Lung Cancer

Cancer of the lung and bronchus is the second most common neoplasm and the leading cause of cancer death in the US, with an estimated 131,880 deaths in 2021 [8].

### 5.1. Current Lung Cancer Screening Recommendations

In 2021, the USPSTF issued a “B” grade recommendation that adults aged 50–80 with a >20 pack-year smoking history, and who smoke currently or have quit within the last 15 years, should receive annual low-dose computer tomography (LDCT). Screening can be discontinued once a patient has not smoked for at least 15 years or they develop a health problem that significantly limits life expectancy. No grade “A” or “C” recommendations were issued [3]. The American Academy of Family Physicians (AAFP) agrees with these recommendations. As of November 2021, the ACS has taken down their 2018 guidelines and has not issued recommendations on lung cancer screening, as it is in the process of reviewing the latest scientific evidence. The National Lung Screening Trial found a 20% reduction in lung cancer mortality with screening. While African Americans have the highest lifetime lung cancer risk (4.4%) and received the greatest reduction in lung cancer mortality with LDCT screening, they were also the least likely group to undergo screening [43]. Some studies have argued that the current USPSTF thresholds for screening are too conservative and disproportionately hurt Black smokers [43,44].

### 5.2. Impact of COVID-19 on Lung Cancer Screening

A retrospective study of a single US institution in Massachusetts found that annual and baseline LDCT volumes were reduced by 72% and 78%, respectively, while follow-up LDCT fell by 50% during the peak COVID period (April 2021). LDCT rates recovered to a 32% decline from baseline by the end of July [45]. According to the *JCO Clinical Cancer Informatics* study, the number of lung cancer diagnoses decreased by 14.9% in March 2020 and 39.1% in April 2020 as compared to 2019. In particular, the rate of new-incidence lung cancer encounters decreased by 23.9% in March 2020 and 46.8% in April 2020 [5]. In contrast, a retrospective hospital-based study out of Italy found only a 2% decrease in lung cancer diagnoses during lockdown [22].

These decreases in screening likely translated to later diagnosis of lung nodules. A prospective observational study using the University of Cincinnati LDCT database found that lung nodules suspicious for malignancy (Lung-RADS 4) after the resumption of routine surgery increased by 21%, and enlarged nodules were increased 31.3% [46]. A prospective modeling study using the National Cancer Registry in England predicted that 5-year survival for lung cancer will decline by 3.5% due to delayed diagnosis [6].

## 6. Other Cancers

The USPSTF no longer recommends universal screening for prostate cancer (PC). Men aged 55–69 have a “C” grade recommendation to undergo prostate-specific antigen (PSA) screening if recommended by their physician due to increased risk or personal priorities. For men aged 70 and older, the USPSTF recommends against screening. The changes in US guidelines have led to significantly fewer false-positive and localized PC diagnoses, and significantly more metastatic PC diagnoses in Caucasian but not African-American men [4]. Likewise, the ACS recommends discussing PSA testing (but not necessarily offering PSA testing) to men aged 50 and above with average risk, men aged 45 and above who are African American or have a first-degree relative diagnosed with prostate cancer below 65, or men aged 40 with multiple first-degree relatives with early PC diagnoses. According to a study of 20 US institutions, PC diagnosis fell by 49.1% in April 2020, exceeded only by the decline in melanoma diagnoses (51.8%) [5].

Screenings for other cancer types are not currently recommended by US societies for the general population. Patients with specific risk factors may be candidates for certain screenings, such as fair skin for melanoma or obesity for endometrial cancer. Patients with genetic conditions or significant family history may also qualify for more intensive screening. Across tumor types, cancer diagnoses decreased by 24.7% in March 2020 and 56.9% in April 2020 in the *JCO Clinical Cancer Informatics* study [5]. Continued follow-up of these databases is required to establish whether cancer screening and diagnosis levels have returned to baseline and how many cancer cases will continue to go undetected due to the pandemic.

## 7. Limitations

This study included a review of screening guideline recommendations from the USPSTF, ACS, AAFP, ACOG and SGO. In the interest of brevity, we did not include recommendations from other US-based professional organizations. Similarly, we compiled and summarized the most-cited retrospective studies on the impact of COVID-19 on cancer screening and diagnosis published in the US and other developed nations as per a Pubmed literature review. Not all studies on this topic were included, and further studies have likely been published since the date of this writing.

## 8. Conclusions

Routine screening for breast, cervical, colorectal, and lung cancers is recommended by the USPSTF and ACS. Prior to COVID-19, rates of screening and early diagnosis in the US were reduced among Black, Hispanic, low socioeconomic status, and rural communities. Alternative at-home screening options like FIT +/− DNA testing for CRC screening have been utilized to successfully reduce these disparities. At the height of the COVID-19 pandemic (April 2020), rates of screening and diagnosis for all cancer types were significantly reduced across the US and around the globe, which is projected to result in later detection and increased mortality. Since lockdown has ended, screening rates have rebounded, but in many geographic areas and for many tumor types they have not yet reached pre-pandemic levels. Greater utilization of at-home testing for CRC has helped alleviate the impact of the pandemic, and self-swab technology for cervical cancer screening has promising results and has become the focus of investigation in the midst of COVID. Outreach efforts that had previously been used to target underprivileged communities are now being employed to restore pre-pandemic rates of screening, though certain populations, such as American Indians and Asian Americans, have experienced the largest and most lasting declines in screening.

## Data Availability

Not applicable.

## Abbreviations

**Acronyms**	
BC	Breast cancer
CRC	Colorectal cancer
PC	Prostate cancer
USPSTF	United States Preventative Services Task Force
ACS	American Cancer Society
NBCCEDP	National Breast and Cervical Cancer Early Detection Program
ACOG	American College of Obstetrics and Gynecology
SGO	Society of Gynecological Oncology
AAFP	American Academy of Family Physicians
hrHPV	High risk human papilloma virus
HSgFOBT	High-sensitivity guaiac fecal occult blood test
FIT	Fecal immunochemical test
LDCTs	Low dose CT scans
PPV, NPV	Positive predictive value, negative predictive value
SEER	Surveillance, Epidemiology, and End Results Program
DBT	Digital breast tomosynthesis
**Screening Guidelines Summarized, USPSTF Grade A or B recommendations included**	
***Breast Cancer***	
USPSTF	Mammography every 2 years age 50–74
ACS	Mammography every year age 45–54, Every 1–2 years age 55-life expectancy <10
***Cervical Cancer***	
USPSTF, ACOG,	Pap smear every 3 years age 21–29,
SGO	Pap + hrHPV testing every 5 years age 30–65
ACS	Pap + hrHPV every 5 years age 25–65
***Colorectal Cancer***	
USPSTF, ACS	Colonoscopy every 10 years age 45–75, alternatively FIT every year, flexible sigmoidoscopy every 5 years, etc.
***Lung Cancer***	
USPSTF, AAFP	LDCT once at age 50–80 for those with a >20 pack years smoking history + current smoker or quit within 15 years
